# SARS-CoV-2 antigenemia and RNAemia in association with disease severity in patients with COVID-19

**DOI:** 10.1038/s41598-024-65489-0

**Published:** 2024-06-28

**Authors:** Dong-Min Kim, Merlin Jayalal Lawrence Panchali, Choon-Mee Kim, Da-Yeon Lee, Jun-Won Seo, Da Young Kim, Na Ra Yun

**Affiliations:** 1 Department of Internal Medicine, College of Medicine, Chosun University, 588 Seosuk-dong, Dong-gu, Gwangju, 501-717 Republic of Korea; 2 Premedical Science, College of Medicine, Chosun University, Gwangju, Republic of Korea

**Keywords:** Antigenemia, Correlation, COVID-19, Mortality, RNAemia, SARS-CoV-2, Diseases, Medical research

## Abstract

Severe acute respiratory syndrome coronavirus 2 (SARS-CoV-2), the virus responsible for COVID-19, causes a spectrum of symptoms ranging from mild upper to severe lower respiratory tract infections**.** However, the dynamics of nucleocapsid (N) protein antigenemia and RNAemia are not fully understood. We conducted a cohort study involving 117 patients with clinically confirmed COVID-19, focusing on the kinetics of antigenemia and RNAemia and their association with various clinical characteristics. The patients had a median age of 66.0 years (52.0–79.0 years), with a gender distribution of 46.2% male and 53.8% female. Antigenemia reached 100% in fatal cases during the first week after admission. The sensitivity/specificity of antigenemia for diagnosis were 64.7%/73.0% at admission, 69.1%/100% in Week 1, and 66.3%/100% in Week 2. Additionally, the rates of antigenemia in asymptomatic patients were 27.3% upon admission and 22.0% in Week 1, respectively; however, no antigenemia was in samples collected in Week 2. Viral RNAemia was not detected in asymptomatic patients, but RNAemia viral loads were elevated in fatal cases. Kaplan–Meier survival curves demonstrated a higher mortality rate when antigenemia concentrations were elevated in the follow-up samples (*P* = 0.005). Our study provides a comprehensive analysis of the kinetics of viral N-protein antigenemia and RNAemia according to disease severity and clinical classification. Our findings suggest that highest concentrations of antigenemia in fatal cases occur in the first week after admission, indicating that early elevated antigenemia may serve as a marker of mortality risk.

## Introduction

Severe acute respiratory syndrome coronavirus 2 (SARS-CoV-2) is the seventh coronavirus known to infect humans since the first report of novel pneumonia (COVID-19) in Wuhan, Hubei Province, China^1^. Coronavirus disease 2019, caused by SARS-CoV-2, exhibits a wide range of clinical manifestations, from mild upper respiratory tract infections to severe lower respiratory tract infections including pneumonia and acute respiratory distress syndrome in several patients^2,3^. Antigenemia, the early detection of antigens in blood, was initially reported for the early diagnosis of cytomegalovirus and to predict the severity of disease proportionately^4^. Similarly, the detection of nucleocapsid antigen (antigenemia) of SARS-CoV-2 in blood has been documented before, but the prognostic implications of antigenemia or RNAemia remain unclear^5^.

Recent studies have highlighted the potential relationship between levels of N-antigenemia and the severity of COVID-19. For instance, research by Chenane et al. revealed that deceased patients exhibited higher median levels of N-antigenemia than surviving patients, particularly in samples collected within the first 8 days after symptom onset^6^. This finding underscores the importance of N-antigenemia as a marker of disease severity. Complementing this, studies by Veyer et al. and Buetti et al. have emphasized the correlation between SARS-CoV-2 RNAemia and severe disease progression, suggesting that the presence of viral RNA in the blood is a strong indicator of critical illness^7,8^. These findings underscore the clinical relevance of monitoring both RNAemia and antigenemia for a better understanding of COVID-19 pathogenesis and for improving patient management strategies. Notably, a higher proportion of SARS-CoV‐2 nucleocapsid antigenemia was found in patients requiring intensive care unit (ICU) admission, suggesting a link between antigenemia and disease severity^9,10^. Interestingly, disease severity may not objectively depend on viral load in respiratory specimens, as both symptomatic and asymptomatic patients presented similar viral loads in respiratory specimens^11^. Despite the clinical significance of viremia in disease progression and the pathogenesis of COVID-19, few studies have examined the importance of RNAemia and antigenemia^12^. The contribution of antigenemia and RNAemia to disease severity and mortality remains poorly understood^10,13–15^.

This study aimed to examine the quantitative dynamics of blood SARS-CoV-2 N protein antigenemia and RNAemia kinetics in 117 patients with clinically confirmed COVID-19. Additionally, we explored the association between antigenemia, RNAemia, and disease severity and mortality.

## Methods

The authors reporting experiments on the use of human and/or human tissue samples were all experiments conducted in accordance with relevant guidelines and regulations. Informed consent was obtained from all subjects and/or legal guardians. The study was conducted in accordance with relevant guidelines and regulations for all methods.

### Participants

In our hospital, we performed a prospective cohort study from June 21, 2020, to September 30, 2023, with patients who possibly had COVID-19 using a preformed case record form. We enrolled 119 patients with confirmed COVID-19 who gave consent for comprehensive use of specimens, admitted from June 21, 2020 to October 22, 2021 at Chosun University Hospital, South Korea. Our aim was to examine the clinical association of antigenemia and RNAemia. All selected patients were clinically confirmed to be SARS-CoV-2 positive using more than one diagnostic methods, including real-time reverse transcriptase polymerase chain reaction (qRT-PCR) with the confirmation of more than two target genes, cell culture, or a ≥ fourfold increase or seroconversion in terms of SARS-CoV-2 antibody titer (enzyme-linked immunosorbent assay [ELISA] or indirect immunofluorescence antibody assay**)**. Moreover, 81 serum/plasma samples of healthy individuals without clinical symptoms, no history of COVID-19, and negative nasopharyngeal qRT-PCR results were used as control samples for the sensitivity assay.

### Sampling and RNA extraction

Peripheral blood was collected from all patients and 200 μL serum/plasma samples from fresh blood were used for ribonucleic acid (RNA) extraction. Concurrently self-collected sputum samples collected from the patients were diluted in phosphate-buffered saline (PBS), mixed by vortexing and pulse-centrifuged for 1 min, and 200 μL supernatant was subjected to RNA extraction. Nasopharynx swabs were directly collected into the commercial UTM™ kits containing 1 mL of a viral transport medium (NobleBio, Oldenzaal, The Netherlands) by a physician, and 200 μL were employed for RNA extraction. The viral RNA was extracted by Real-prep Viral DNA/RNA Kit (Biosewoom, Seoul, South Korea) using a fully automated instrument (Real-Prep system, Biosewoom).

### Real-time reverse transcription-polymerase chain reaction (qRT-PCR) for SARS-CoV-2 detection

For the qRT-PCR assay of the nucleocapsid protein (*NP*) gene, primers and probes were designed in-house, nCov-NP_572F (5′-GCAACAGTTCAAGAAATTC-3′), nCov-NP_687R (5′-CTGGTTCAATCTGTCAAG-3′), and nCov-NP_661P (5′-FAM-AAGCAAGAGCAGCATCACCG-BHQ1-3′). Thermal cycling was performed as follows: 50 °C for 10 min for reverse transcription, one cycle of 95 °C for 30 s for preincubation, 95 °C for 5 s at 57 °C for amplification, and 45 cycles for data detection. For the target genes* E* (encoding envelope protein) and *RdRp* (encoding RNA-dependent RNA polymerase), the Kogene Kit (Kogene Biotech Co., Ltd., Seoul, South Korea) and SD Kit (SD Biotechnologies Co., Ltd., Seoul, South Korea) were used, and amplification was performed according to the manufacturer’s instructions. For the *NP* target, qRT-PCR was performed in an Exicycler™ 96 Real-Time Quantitative Thermal Block (Bioneer, Smiths Parish, Bermuda), and for Kogene and SD kits, the CFX96 Touch™ Real-Time PCR Detection System (Biorad, Hercules, CA, USA) was used. Cycle threshold (C_t_) values were set to ≤ 40 for the reference gene and were assumed to denote a positive result.

### Cell culture

For the identification of SARS-CoV-2 in culture, monolayers of Vero E6 cells were cultured in Dulbecco’s modified Eagle’s medium supplemented with 10% of fetal bovine serum and a 1 × penicillin–streptomycin antibiotic solution (Gibco, Thermo Fisher Scientific, Waltham, MA, USA) in an atmosphere containing 5% of CO_2_ at 37 °C. Then, 200 μL of an unfrozen swab sample in viral transport medium (UTM™ kit, NobleBio) diluted with 1 mL of Dulbecco’s phosphate-buffered saline (Welgene, Taipei, Fujian, China) was inoculated to the monolayer of cultured Vero cells. After two passages, viral proliferation was confirmed by qRT-PCR with a confirmatory C_t_ value < 20 or an indirect immunofluorescence assay using in-house SARS-CoV-2 antigen slides. Meanwhile, inoculated cells were examined daily for cytopathic effects, as described for SARS-CoV and MERS-CoV in other studies^16,17^.

### Sandwich ELISA for antigenemia

The nucleocapsid protein (N) antigenemia assay of patients with and without COVID-19 was carried out using single molecule array (SIMOA) technology with paramagnetic microbeads–based sandwich ELISA. The SIMOA SARS-CoV-2 N Protein Advantage kit assay (Quanterix Corp, Boston, MA, USA, PN/103806) is a digital immunoassay that quantitatively measures the SARS-CoV-2 nucleocapsid protein in human serum and plasma. Plasma or serum obtained from fresh blood was frozen after aliquoting to minimize protein degradation due to freeze–thaw cycles and thawed at room temperature before use for antigenemia assay. Briefly, each well of 96-well ELISA microplates (Quanterix® plates) was loaded with 4 × dilution of plasma or serum and assayed in Simoa HD-X instrument (Quanterix) using a two‐step immunoassay. For detection, incubation was performed with the target antibody coated with paramagnetic beads, sample, and biotinylated antibody (SIMOA Guide Quanterix). The nucleocapsid protein present in the sample was captured using antibody-coated beads bound to the biotinylated antibody, and detected simultaneously as described previously^18,19^.

### Indirect ELISA

Indirect ELISA for SARS-CoV-2 was performed using a recombinant nucleocapsid protein (Bioapp. Inc., Pohang, South Korea) to determine serological titers of immunoglobulin G (IgG), immunoglobulin M (IgM), and total antibodies. Frozen serum samples were thawed at room temperature and used for indirect ELISA. In brief, 100 µL of 2 µg/mL recombinant SARS-CoV-2 nucleocapsid protein (Bioapp. Inc.) was coated in a 96-well ELISA microplate (Thermo Fisher Scientific) with carbonate-bicarbonate buffer, with overnight incubation at 4 °C. The ELISA plates were washed with PBS containing 0.05% Tween 20, followed by 2-h blocking at 37 °C with 5% skim milk in blocking buffer. The plates were further washed incubated for another 2 h at 37 °C with the serum samples diluted 100-fold in blocking buffer. After washing, a secondary antibody (horseradish peroxidase-conjugated goat anti-human IgG antibody [1:6000, Invitrogen, Thermo Fisher Scientific, Cat A18805], anti-human IgM antibody [1:3000, Invitrogen, Thermo Fisher Scientific, Cat 31415], or anti-human total-antibody antibody [1:40,000; Thermo Fisher Scientific, Cat 31418]) was added, and incubated again for another 1 h at 37 °C. The plates were further washed and 50 µL of the 3,3′5,5’-tetramethylbenzidine substrate (Sigma-Aldrich, St. Louis, MO, USA) was added and incubated at room temperature (20–30 °C) for 30 min in dark. Moreover, 25 µL of 1 M H_2_SO_4_ was added for arresting the reaction and the optical density was measured using an Epoch™ two microplate spectrophotometer (Kitchener, ON, Canada) at 450 nm (OD_450_). The cutoff values and positivity for SARS-CoV-2 were set as described previously^20^.

### Statistical methods

Statistical analyses were performed using MedCalc 20·013 software (Ostend, Belgium), and IBM SPSS Statistics for Windows, version 26.0. (IBM Corp., Armonk, NY, USA), and GraphPad Prism 9 (San Diego, CA, USA). The sensitivity, specificity, and accuracy of the test were evaluated using receiver operating characteristic (ROC) curve analysis. Confidence intervals for sensitivity, specificity and accuracy are “exact” Clopper–Pearson confidence intervals^21^. To determine the 40-day survival rate, Kaplan–Meier survival analysis was conducted based on the antigenemia concentration. Quantitative variables are presented as mean ± standard deviation and n (%) for normally distributed variables. Mean values were compared using t-tests for continuous variables and were found to be normally distributed. *P*-values comparing patients with COVID-19 with evidence of antigenemia and RNAemia to those without antigenemia and RNAemia were calculated using the Mann–Whitney U or Fisher’s exact test, as appropriate.

## Results

### Clinical and demographic characteristics of patients with COVID-19

This study included 119 patients with clinically confirmed COVID-19 who were admitted and treated at Chosun University Hospital, Gwangju, South Korea, from June 2020 to December 2021. The median age of patients was 66.0 years (interquartile range: 52.0–79.0 years) while the median ages of the survival and non-survival groups were 62.0 years (48.5–74.3 years) and 83.0 years (79.0–89.0 years), respectively. The distribution of patients by gender was 46.2% male and 53.8% female. Among those with underlying comorbidities, 38.7% had hypertension, followed by 25.2% with diabetes mellitus; approximately 64.7% had some underlying comorbidities. Detailed characteristics are presented in Table 1. Upon admission, several patients exhibited symptoms, such as fever (37.0%), cough (34.5%), headache (7.6%), chills (16.0%), sore throat (15.1%), and myalgia (13.4%). Regarding treatment, 51.3% of patients received supplemental oxygen, 27.7% were given high-flow oxygen, and 16.8% required mechanical ventilation; 47.1% received antiviral treatment and 36.1% underwent steroid therapy. Additionally, approximately 83.2% of patients exhibited antigenemia in their peripheral blood, with 27.7% having elevated antigenemia; the fatal group displayed the highest percentage of elevated antigenemia (64.7%). As shown in Table 1, about 23.5% of patients had RNAemia, with 76.5% of the cases being fatal.

**Table 1:** Clinical characteristics of COVID-19 patients.

Characteristics	Total (N = 119)		Survival group (n = 102)		Fatal group (n = 17)		*P* value
	n	%	n	%	n	%	
Male gender	55	46.2%	49	48.0%	6	35.3%	0.329
Age, median (IQR)	66.0	52.0–79.0	62.0	48.5–74.3	83.0	79.0–89.0	** < 0.001**
Antigenemia	99	83.2%	83	81.4%	16	94.1%	0.001
No elevation	67	56.3%	63	61.8%	4	23.5%	
Elevation	33	27.7%	22	21.6%	11	64.7%	
Unknown of elevation	19	16.0%	17	16.7%	2	11.8%	
Antigenemia peak, Mean ± SD	119	463.36 ± 1566.10	102	342.31 ± 1219.67	17	1189.65 ± 2836.22	0.242
Antigenemia peak, median(IQR)	119	26.40 (3.51–148.0)	102	25.75 (1.51–157.0)	17	35.70 (13.05–694.15)	0.242
RNAemia during hospitalization	28	23.5%	15	14.7%	13	16.5%	** < 0.001**
RNAemia on admission	21	17.6%	10	9.8%	11	64.7%	** < 0.001**
Nasopharyngeal viral load on admission, Ct value (mean ± SD)	119	22.58 ± 8.632	102	23.38 ± 8.876	17	17.80 ± 4.884	**0.001**
Nasopharyngea viral load on admission, Ct value [median(IQR)]	119	21.01 (15.23–29.15)	102	22.22 (15.91–30.71)	17	18.38 (13.79–20.88)	**0.001**
Comorbidities, N (%)	77	64.7%	62	60.8%	15	88.2%	**0.028**
Cardiovascular disease	16	13.4%	14	13.7%	2	11.8%	1.000
Diabetes mellitus	30	25.2%	22	21.6%	8	47.1%	**0.035**
Hypertension	46	38.7%	36	35.3%	10	58.8%	*0.065*
Chronic lung disease	3	2.5%	1	1.0%	2	11.8%	*0.053*
Cancer	8	6.7%	7	6.9%	1	5.9%	1.000
Chronic kidney disease	2	1.7%	2	2.0%	0	0.0%	1.000
Severity PSI*, mean ± SD	66.32	24.930	62.44	23.453	92.92	17.863	** < 0.001**
Symptoms							
Fever	44	37.0%	38	37.3%	6	35.3%	0.877
Cough	41	34.5%	35	34.3%	6	35.3%	0.937
Headache	9	7.6%	9	8.8%	0	0.0%	0.355
Chill	19	16.0%	19	18.6%	0	0.0%	*0.071*
Sore throat	18	15.1%	17	16.7%	1	5.9%	0.464
Myalgia	16	13.4%	16	15.7%	0	0.0%	0.123
Treatments							
Oxygen inhalation	61	51.3%	44	43.1%	17	100.0%	** < 0.001**
High flow oxygen therapy	33	27.7%	17	16.7%	16	94.1%	** < 0.001**
Mechanical ventilation	20	16.8%	7	6.9%	13	76.5%	** < 0.001**
Antiviral	56	47.1%	40	39.2%	16	94.1%	** < 0.001**
Steroids	43	36.1%	28	27.5%	15	88.2%	** < 0.001**

*Data are expressed as the mean ± SD or N (%). The data included 117 patients who participated in the study. Missing data, n = 17 (survival 13 / fatal 4).

*N* number of patients, *%* percentage, *SD* standard deviation.

Significant values are given in bold and italics.

### Biochemical and laboratory characteristics of patients with COVID-19 on admission

The biochemical and laboratory diagnostic characteristics of survival and fatal cases were determined. The median white blood cell (WBC) counts for survival and fatal cases were 5.8 × 10^9^/L and 6.4 × 10^9^/L, respectively, with elevated WBC observed in fatal cases. Similar trends were noted for other biochemical and laboratory findings. Conversely, a decrease in the lymphocyte count was noted in fatal cases. Detailed biochemical and laboratory findings are summarized in Table 2.

**Table 2:** Laboratory findings of COVID-19 patients on admission.

Characteristics	Total (N = 119)			Survival group (n = 102)			Fatal group (n = 17)			*P* value
	n	median	IQR	n	median	IQR	n	median	IQR	
WBC (10^3^ /mL)	118	5.9	4.5–7.2	101	5.8	4.4–7.0	17	6.4	4.9–8.5	0.201
Neutrophils (%)*	118	70.15	± 14.22	101	68.08	± 13.77	17	82.45	± 10.26	** < 0.001**
Lymphocytes (%)*	118	21.32	± 11.77	101	22.87	± 11.63	17	12.08	± 7.79	** < 0.001**
Monocyte (%)*	118	7.37	± 3.41	101	7.75	± 3.32	17	5.12	± 3.15	**0.003**
Eosinophil (%)	118	0.3	0.0–1.5	101	0.5	0.1–1.8	17	0.0	0.0–0.0	** < 0.001**
Hb (g/dL)*	117	13.06	± 1.69	101	13.24	± 1.64	17	12.05	± 1.60	*0.006*
PLT	118	206.0	158.0–244.0	101	212.0	170.5–255.5	17	134.0	122.5–184.0	< *0.001*
CRP (mg/dL)	105	1.7	0.2–7.1	88	0.8	0.2–6.2	17	9.3	3.6–19.9	< *0.001*
Troponin-I (ng/mL)	79	0.0	0.0–0.0	64	0.0	0.0–0.0	15	0.0	0.0–0.1	** < 0.001**
AST (U/L)	118	26.4	18.7–44.1	101	24.1	18.1–40.9	17	56.8	30.0–67.3	**0.003**
ALT (U/L)	118	20.6	12.0–30.5	101	20.7	12.0–30.8	17	17.6	12.5–29.0	0.893
Creatinine (mg/dL)*	118	0.97	± 1.24	101	0.96	± 1.32	17	1.05	± 0.56	0.790
Procalcitonin (ng/mL)	103	0.1	0.0–0.1	86	0.0	0.0–0.1	17	0.2	0.1–0.7	**0.001**
Fibrinogen (mg/dL)*	82	405.27	± 120.06	68	401.84	± 117.02	14	421.93	± 137.37	0.572
D-dimer	**85**	282.0	153.0–524.0	70	226.0	139.5–474.8	15	478.0	299.0–885.0	**0.002**
NLR	118	3.6	1.9–6.5	101	3.1	1.8–6.0	17	6.3	4.0–18.1	** < 0.001**
CK-MB (ng/mL)	101	1.4	0.9–2.5	84	1.4	0.8–2.1	17	2.3	1.4–5.2	**0.007**
Potassium (mEq/L)	115	3.9	3.6–4.2	98	3.9	3.6–4.2	17	4.2	3.8–4.6	***0.066***

*N* number of patients, *L* liter, *WBC* white blood cell, *Hb* hemoglobin, *CRP* C-reactive protein, *AST* aspartate aminotransferase, *ALT* alanine aminotransferase.

*Described by mean ± SD.

Significant values are given in bold, italics, bold italics.

### Assessment of viral antigenemia in serum/plasma samples

**Figure 1:**
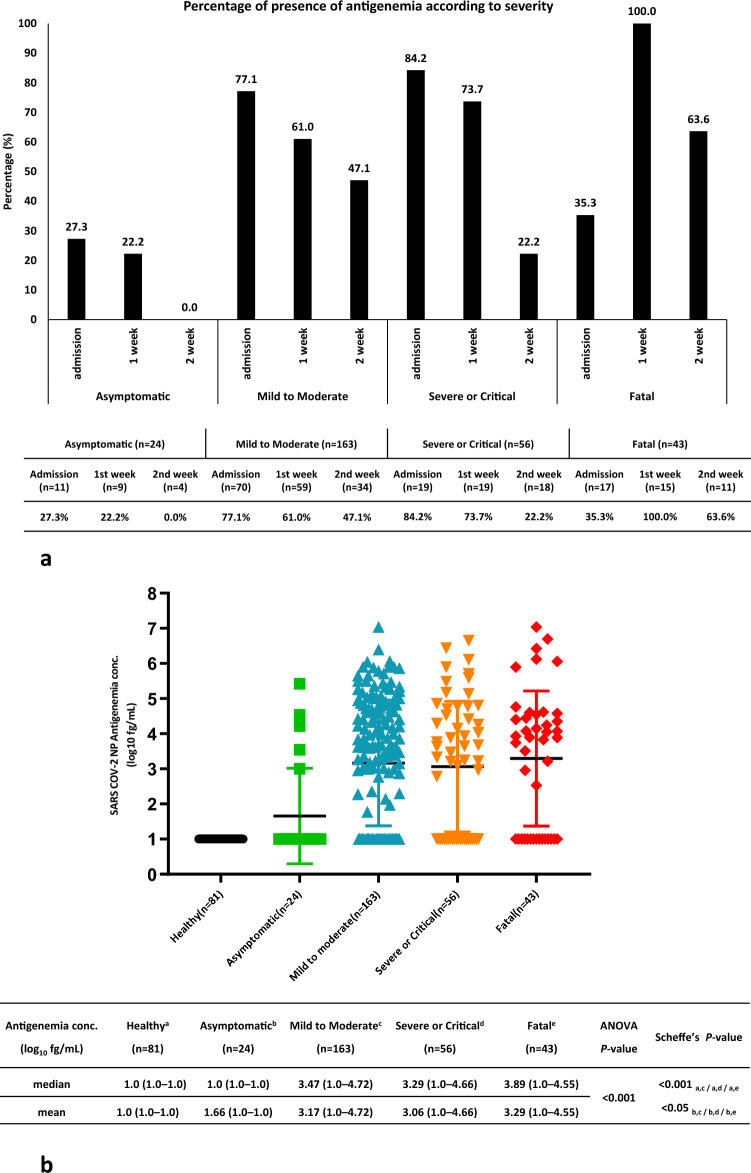
(**a**) Direct percentage and proposition of viral antigenemia according to disease severity. Patient samples were classified according to the Sixth Revised Trial Version of the Novel Coronavirus Pneumonia Diagnosis and Treatment Guidance, and the data are expressed as N (%). (**b**) Concentration of antigenemia according to disease severity. Data are expressed as means and medians. *P* < 0.05 was considered statistically significant.

Patients were categorized into four subgroups: asymptomatic, mild to moderate, severe or critical, and fatal, according to the Sixth Revised Trial Version of the Novel Coronavirus Pneumonia Diagnosis and Treatment Guidance^22^. A total of 364 serum/plasma samples from 119 patients with COVID-19 and 81 healthy individuals were assayed for the presence of viral nucleocapsid protein antigenemia of SARS-CoV-2 using a sandwich ELISA. The percentage of antigenemia on admission and in the first week in asymptomatic patients was 27.3 and 22.2%, respectively. However, no antigenemia was observed in the second week in asymptomatic patients (Fig. 1a). In the mild to moderate category, the antigenemia rates on admission, Week 1, and Week 2 were 77.1, 61.0, and 47.1%, respectively. Similarly, the proportion of antigenemia in severely ill or critical patients was 84.2, 73.7, and 22.2% on admission, Week 1, and Week 2, respectively. Interestingly, the proportion of antigenemia in fatal patients with COVID-19 was low on admission (35.3%), peaking at 100.0% in Week 1 samples and decreasing to 63.6% in Week 2 (Fig. 1a**)**. We attempted to measure the antigenemia concentration according to the classified patients, where the median concentration of N-protein antigen was below 1 log_10_ fg/mL in both healthy and asymptomatic patients. In contrast, for mild to moderate, and severe or critical cases, the antigenemia levels were high with a median of 3.47 and 3.29 log_10_ fg/mL respectively, but in fatal cases, the concentration was the highest with a median of 3.89 log_10_ fg/mL. Therefore, we assayed the mean distribution of antigenemia using one-way analysis of variance (ANOVA) followed by post hoc analysis (Scheffé’s test) and found that the antigenemia levels were significantly different according to disease severity (*P* < 0.001; Fig. 1b).

### Analysis of specificity and sensitivity of antigenemia and upper and lower respiratory tract specimens

**Figure 2:**
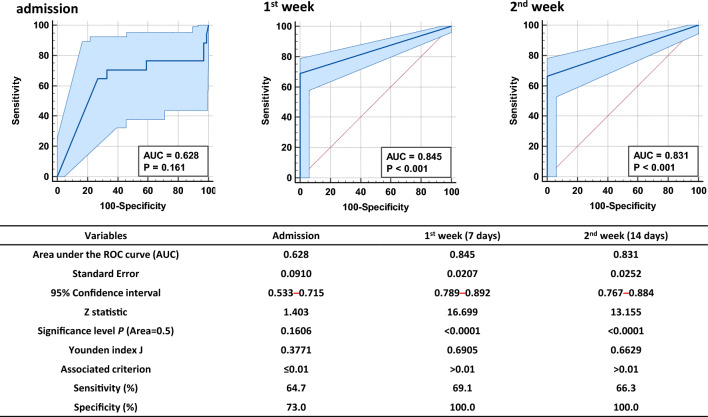
Specificity and sensitivity of antigenemia. (**a**) Determination of specificity and sensitivity in admission samples for antigenemia. (**b**) Determination of specificity and sensitivity on Week 1 samples for antigenemia. (**c**) Determination of specificity and sensitivity on Week 2 samples for antigenemia. (**d**) The data are presented with ROC curve. *P-*values < 0.05 indicate significant differences.

To examine the accuracy of the results, we evaluated the sensitivity and specificity using the area under the ROC curve. The sensitivity and specificity of admission sample antigenemia were 64.7 and 73.0%. As expected, the sensitivities of antigenemia on Weeks 1 and 2 were 69.1 and 66.3%, and the specificity was 100.0% (Fig. 2). The sensitivity of antigenemia was assayed based on the time interval from the day of symptom onset to the second week post symptom onset. Our results clearly demonstrated that the first week of infection had the highest sensitivity of antigenemia, which drastically reduced after the second week. To further confirm the sensitivity of antigenemia and viral load of the upper and lower respiratory tracts of admission samples, an ROC curve comparison was performed, and our results were validated and found to be statistically significant (Supplementary Fig. S1). RT-qPCR targeting the *E* and *RdRp* genes using the Kogene Kit (Kogene Biotech) was performed to assay the sensitivity of the upper (nasopharyngeal) and lower (sputum) respiratory tract specimens of all patients with antigenemia according to the time interval from symptom onset to the recovery phase. Furthermore, while correlating the antigenemia results with both upper and lower respiratory tract viral loads, the sensitivity began to decrease in the second week. The results are summarized in Table 3.

**Table 3:** Sensitivity of antigenemia and in upper and lower respiratory-tract specimens at different time point.

Symptom onset	COVID-19 Antigenemia				Upper respiratory track E gene(Kogene kit)				Lower respiratory track E gene (Kogene kit)			
	Positive	Negative	Total	Sensitivity	Positive	Negative	Total	Sensitivity	Positive	Negative	Total	Sensitivity
− 3 to 0	17	6	23	73.9	9	2	11	81.8	9	0	9	100.0
1 to 3	29	4	33	87.9	14	1	15	93.3	8	2	10	80.0
4 to 6	18	9	27	66.7	17	4	21	80.9	9	5	14	64.3
7 to 9	35	13	48	72.9	11	6	17	64.7	13	1	14	92.9
10 to 12	21	9	30	70.0	14	2	16	87.5	6	7	13	46.2
13 to 15	28	13	41	68.3	10	3	13	76.9	8	4	12	66.7
16 to	20	37	57	35.1	16	38	54	29.6	22	31	53	41.5

Samples were segregated from the admission date with 3 days interval until 16 + days; cycle threshold (cutoff) of 35; C_t_-35 was used according to the manufacturer’s instructions. Nasopharyngeal and lower respiratory tract sputum samples were collected from the upper respiratory tract.

### Assessment of viral RNAemia in serum/plasma samples

**Figure 3:**
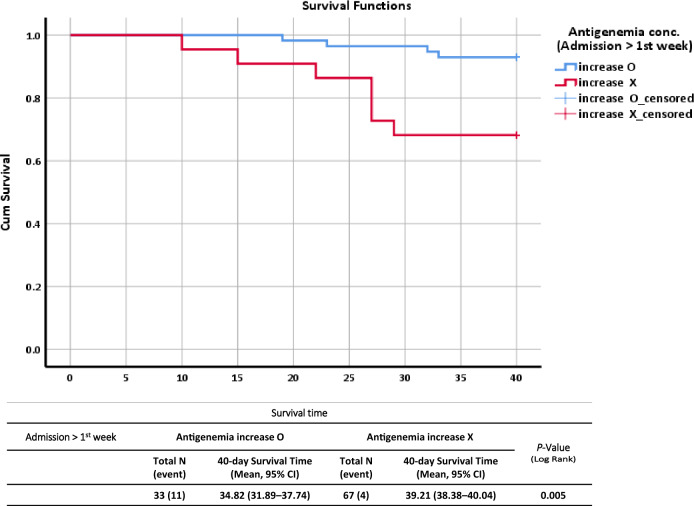
Kaplan–Meier curve for mortality, plotted using antigenemia of patient samples from admission to Week 1. A 40-day survival time was set for all patients without mortality. *‘*O’ represents an increase in antigenemia, ‘X’ represents a stable or decreased antigenemia concentration, and ‘Event’ represents mortality*. P*-values comparing patients with COVID-19 showing evidence of increased antigenemia were calculated using the Mann–Whitney U test or Fisher’s exact test, as appropriate. *P-*values < 0.05 indicate significant differences.

SARS-CoV-2 viral RNA in blood on admission and in the first and second week samples was assayed using RT-qPCR targeting the nucleocapsid gene. All samples, including those from healthy, asymptomatic, mild to moderate, severe or critical, and fatal cases, were assessed using RT-qPCR to identify the presence of RNAemia. Viral RNAemia was not detected in asymptomatic patients during the entire study period, and none of the samples collected in the second week showed RNAemia (Table 4). Similarly, antigenemia and RNAemia concentrations in the plasma samples of fatal cases were substantially higher in both the admission and first week samples than in the other groups of patients. Moreover, ANOVA, followed by the Scheffé post hoc criterion, for the viral load of RNAemia and antigenemia in different categories of patients was performed, and was statistically significant (*P* < 0.001) for the admission and first week samples (Table 4). The survival of patients with antigenemia is presented using Kaplan–Meier curves in Fig. 3. Our results predicted a higher mortality rate in patients with elevated concentrations of antigenemia in follow-up samples than in admission samples. Hence, as shown in Fig. 3, our results clearly demonstrated that elevated antigenemia in the first week after admission in fatal cases was more likely to be a severity marker of mortality.

**Table 4:** Time-dependent analysis of RNAemia, antigenemia, and antibody responses (IgM, IgG, and Total Ab) across different clinical severities of COVID-19 patients.

	Severity	RNAemia			Antigenemia			Antibody using ELISA								
								IgM			IgG			Total Ab		
		N (%)	ANOVA P value	Scheffe’s P value	N (%)	ANOVA P value	Scheffe’s P value	N (%)	ANOVA P value	Scheffe’s P value	N (%)	ANOVA P value	Scheffe’s P value	N (%)	ANOVA P value	Scheffe’s P value
Admission	Asymptomatic^a^ (N = 11)	0 (0%)	< 0.001	< 0.001a,d/b,d 0.002b,c 0.027a,c	3 (27.27%)	< 0.001	0.006b,d 0.007a,b 0.009a.c 0.011c,d	5 (45.4%)	0.773		5 (45.4%)	0.922		5 (45.4%)	0.567	
	Mild to Moderate^b^ (N = 70)	3 (4.29%)			54 (77.14%)			25 (35.7%)			25 (35.7%)			25 (35.7%)		
	Severe or critical^c^ (N = 19)	7 (36.84%)			16 (84.21%)			4 (21.1%)			4 (21.1%)			4 (21.1%0		
	Fatal^d^ (N = 17)	11 (64.71%)			6 (35.29%)			5 (29.4%)			5 (29.4%)			5 (29.4%)		
1 week	Asymptomatic^a^ (N = 9)	0 (0%)	0.059		2 (22.22%)	0.001	0.047a,c 0.001a,d 0.031b,d	5 (55.6%)	0.021	NA	5 (55.6%)	0.499		5 (55.6%)	0.159	
	Mild to Moderate^b^ (N = 59)	4 (6.78%)			59 (100%)			23 (39%)			23 (39%)			22 (37.3%)		
	Severe or critical^c^ (N = 19)	1 (5.26%)			14 (73.68%)			3 (15.8%)			3 (15.8%)			3 (15.8%)		
	Fatal^d^ (N = 15)	4 (26.67%)			15 (100%)			4 (26.7%)			4 (26.7%)			4 (26.7%)		
**2 week**	Asymptomatic^a^ (N = 4)	0 (0%)	NA		0 (0%)	0.039		2 (50%)	0.104		2 (50%)	0.775		2 (50%)	0.441	
	Mild to Moderate^b^ (N = 34)	0 (0%)			16 (47.06%)		NA	13 (38.2%)			13 (38.2%)			13 (38.2%)		
	Severe or critical^c^ (N = 18)	0 (0%)			4 (22.22%)			3 (16.7%)			3 (16.7%)			3 (16.7%)		
	Fatal^d^ (N = 11)	0 (0%)			7 (63.64%)			2 (18.2%)			2 (18.2%)			2 (18.2%)		

Data are expressed as mean ± SD or N (%). *N* number of patients, *NA* not applicable. The first sample was collected on admission, first week follow-up sample was collected from the 5th to 9th day of admission, second week- sample was collected from the 12th to 16th day of admission. RNAemia, Antigenemia, and Antibody response comparisons among multiple subgroups were performed using one-way analysis of variance (ANOVA) followed by Scheffe's post hoc test. ^a,b,c,d^ represents the categories of patients. *P < 0.05, statistical significance.

### Assessment of IgG, IgM, and total antibody response in serum samples

We conducted in-house indirect ELISA using plant-expressed recombinant nucleocapsid protein to measure IgG, IgM, and total antibody responses against SARS-CoV-2 in serum samples collected on admission, in the first week, and in the second weeks. The positive cutoff values for IgG, IgM, and total antibody responses were established using the mean plus three standard deviations from 1:100 diluted serum samples of 20 healthy individuals^20^. We compared the IgM, IgG, and total antibody levels across different patient groups (asymptomatic, mild to moderate, severe or critical, and fatal) and at various time points during hospital admission. ANOVA analysis revealed no significant differences in IgG and total antibody values either between the groups or over time. However, significant differences in IgM levels were observed among the groups during the first week, although Scheffe's post hoc test indicated no significant differences (Table 4).

### Correlation of antigenemia and RNAemia in disease severity

**Figure 4:**
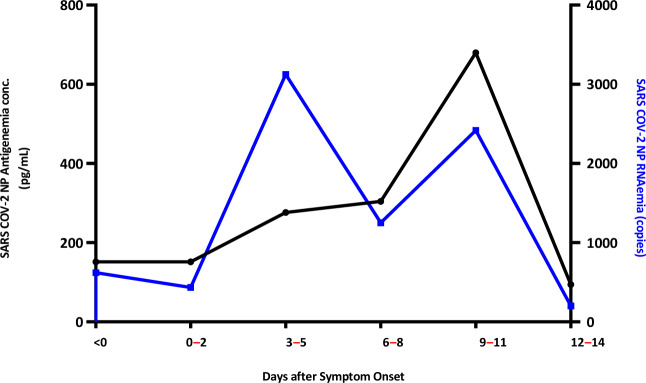
Antigenemia concentration and RNAemia viral copy number over time. The concentrations of antigenemia and RNAemia viral copy number are presented as mean ± standard deviation. Samples were assayed from admission to Week 2 post symptom onset.

**Figure 5:**
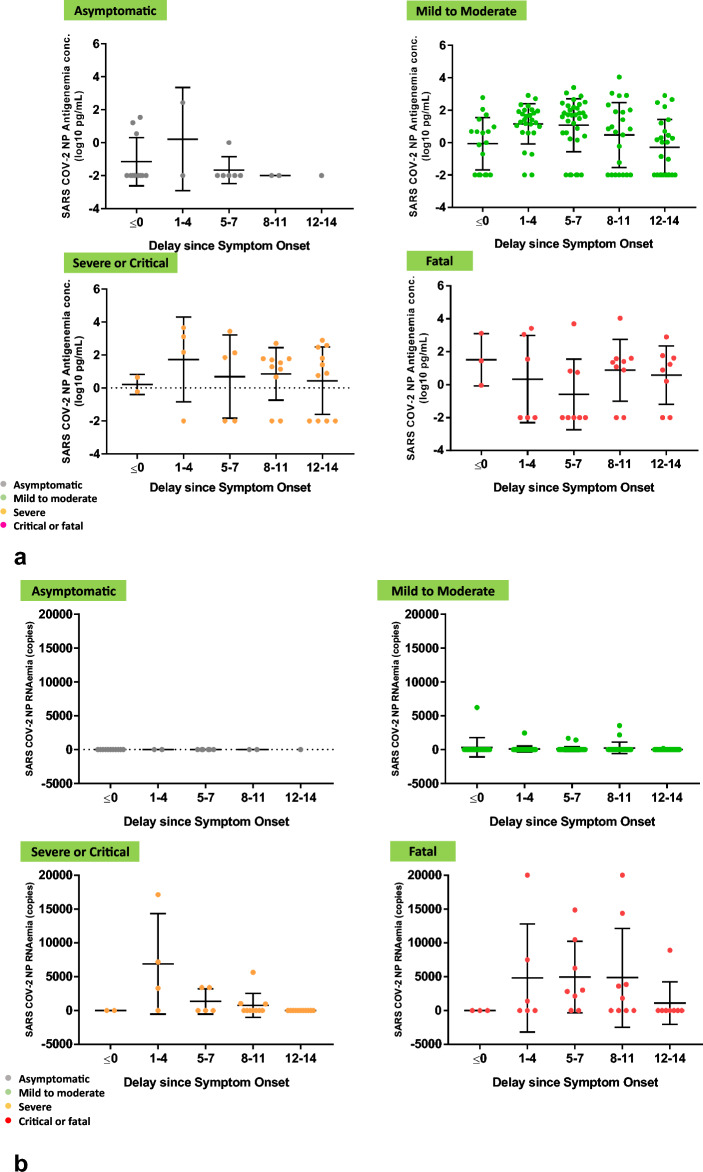
(**a**) Antigenemia concentration (pg/mL) according to disease severity at different time point. (**b**) RNAemia copy number according to disease severity at different time points. Patient samples were classified according to the Sixth Revised Trial Version of the Novel Coronavirus Pneumonia Diagnosis and Treatment Guidance. Samples were assayed from admission to Week 2 post symptom onset. The data are expressed as mean ± standard deviation.

To further confirm the correlation between antigenemia and RNAemia, we examined the relationship between antigenemia concentration and RNAemia viral load in accordance with the SARS-CoV-2 disease category at various time points. The relationship between antigenemia and RNAemia viral copy is presented at different time intervals in Fig. 4. Our results show an early peak in RNAemia at 3–5 days followed by a decrease; however, for antigenemia, the peaks were observed at approximately 9–11 days. We further analyzed both antigenemia and RNAemia according to asymptomatic, mild to moderate, severe or critical, and fatal conditions in accordance with the time interval. The dynamics of viral antigenemia clearly demonstrated that in asymptomatic patients, the presence of antigenemia sharply decreased in the first week, and in other categories, the concentration of antigenemia was sustained for a longer period, as shown in Fig. 5a. Furthermore, considering viral RNAemia kinetics, no RNAemia was found in any of the asymptomatic patients. However, in mild to moderate cases, a decreasing pattern was observed in accordance with the time interval. In contrast, in the fatal cases, RNAemia was sustained until Week 2 (Fig. 5b). Hence, our results clearly showed that RNAemia and antigenemia were directly correlated with disease severity, specifically in fatal cases where elevated antigenemia and RNAemia were observed for a prolonged time.

## Discussion

Our study elucidated significant correlations between clinical outcomes and the dynamics of SARS-CoV-2 RNAemia and antigenemia among COVID-19 patients. We observed that both RNAemia and antigenemia concentrations were significantly higher in fatal cases compared to other severity groups, particularly during the first week post-admission. This timeframe emerges as critical for prognostication, with elevated antigenemia potentially serving as an early indicator of mortality. Notably, RNAemia, absent in asymptomatic individuals, peaked early (3–5 days after symptom onset) before declining, yet persisted in severe and fatal cases. This persistence underscores the potential of RNAemia as a marker of disease severity.

Despite the clinical significance of viremia in the progression of COVID-19 and its outbreaks, few studies have focused on the importance of RNAemia and antigenemia. While several recent investigations have highlighted a potential correlation between N-antigenemia levels and the severity of COVID-19, the relationship between SARS-CoV-2 nucleocapsid protein antigenemia in the blood and its association with disease severity and fatal outcomes remains poorly understood. Similarly, the correlation and kinetic comparison between antigenemia and viral RNAemia have not been extensively studied. In a previous study on human cytomegalovirus infections, monitoring of pp65 antigenemia was compared with the results of quantitative PCR of the nucleic acids^23^. In an asymptomatic patient with SARS-CoV-1 in 2004, antigenemia and seroconversion were well documented^24^. In another case study, persistent antigenemia and RNAemia were observed for an extended period post-symptom onset^25^. Furthermore, a previous study reported that SARS-CoV-2 viral N-antigenemia and RNAemia were independently associated with fatal clinical outcomes in ICU patients only^26^. Antigenemia, RNAemia, and various RT-PCR assays are widely used to monitor SARS-CoV2 viral infections globally. Given the various clinical and severity markers of SARS-CoV2, studying the sensitivity and comparison of these markers, as well as their clinical usefulness in the diagnosis and prediction of disease severity, is important.

In this study, we comprehensively analyzed the kinetics of viral N-protein antigenemia in patients with COVID-19 according to disease severity, clinical classification, and time dependence. Despite numerous studies focusing on hematological and biochemical biomarkers to identify risk factors for COVID-19 mortality, data on antigenemia as a potential key biomarker remain sparse^27^. To address this gap, we compared the hematological and biochemical profiles between the COVID-19 survival group (n = 102) and the death group (n = 17). In the death group, we observed elevated levels of WBC, troponin-I, D-dimer, and CK-MB, alongside decreased lymphocytes and eosinophils, aligning with findings reported by Henry et al. in their hematological, biochemical, and immunological analyses^27^. Building on this, our investigation into the kinetics of viral antigenemia provided robust evidence that antigenemia peak during the first week after symptom onset and declines in the second week. Furthermore, we demonstrated a potential relationship between elevated antigenemia concentration and RNAemia in mortality outcomes. We further confirmed that the antigenemia concentration and RNAemia viral load were elevated in severe-to-critical patients; however, both antigenemia and RNAemia had the highest concentrations in fatal cases. To our knowledge, this is the first study to report that fatal cases have the highest antigenemia concentrations in first week after admission.

The statistically significant correlation between elevated levels of antigenemia and RNAemia, particularly in the first week post-admission in fatal cases, indicates these markers can be critical in predicting patient outcomes. The Kaplan–Meier curves, demonstrating a higher mortality rate among patients with elevated antigenemia levels, further validate the prognostic value of these viral components. This correlation between early elevated antigenemia and mortality risk suggests that interventions aimed at reducing viral load or mitigating its effects may be most beneficial if initiated promptly upon hospital admission. Our study notably confirmed the prolonged persistence of N antigenemia and high levels of viral replication in deceased patients. We believe that these phenomena result from multiple factors, including underlying health conditions, the host immune response, and SARS-CoV-2 viral characteristics. Consequently, our results underscore the importance of early detection and intervention, especially for patients at high risk for severe outcomes. In our study, the fatal group of COVID-19 patients had underlying comorbidities (*P* = 0.028), such as diabetes mellitus (*P* = 0.035), hypertension (*P* = 0.065) and chronic lung disease (*P* = 0.053) as indicated in Table 1. According to Guan W-J et al., patients with comorbidities exhibited poorer clinical outcomes and greater disease severity compared to those without comorbidities^28^. These health conditions can lead to immunosuppression, making it challenging for the body to mount an effective response against the virus and thereby facilitating high levels of viral replication. In severe COVID-19 cases, the immune system's failure to efficiently eliminate the virus can result in prolonged viral replication and the presence of antigens. The consistently high levels of viral replication in deceased patients likely contribute to the prolonged presence of viral antigens. Notably, fatal cases had significantly higher concentrations of N protein antigenemia, especially in the first week post-admission, which could reflect an overwhelming viral replication beyond the host's control. Furthermore, SARS-CoV-2 variants, with mutations in the spike protein and other regions, may exhibit differences in infectivity and immune escape potential.

This study had some limitations. Firstly, it was conducted with a single cohort at Chosun University Hospital in Gwangju, South Korea, potentially limiting the applicability of our findings to a wider population. Second, not all samples underwent viral culture, which might have provided additional insights into the dynamics of viral replication. Despite these limitations, our findings provide valuable information on the prognostic significance of viral markers such as RNAemia and antigenemia in the early stages of COVID-19 infection. However, these insights indicate the need for further, more comprehensive studies. Future research should include more diverse patient groups and explore additional biomarkers that could influence the severity and mortality of COVID-19.

In conclusion**,** we comprehensively analyzed the kinetics of viral N-protein antigenemia and other clinical factors in accordance with disease severity, clinical classification, and time dependency in patients with COVID-19. We found that antigenemia concentrations are highest during the first week following admission in fatal cases, suggesting that elevated levels of antigenemia during this critical period could serve as an important marker of disease severity and a predictor of mortality. These findings underscore the potential of using antigenemia as an early indicator for severe outcomes in COVID-19 patients.

## Ethical approval

Approval for this study was granted by the Chosun University Hospital. Institutional Review Board (2020-04-003).

### Supplementary Information

Supplementary Figure 1.

## Data Availability

All data generated or analysed during this study are included in this published article [and its supplementary information files].
