# Supplementary material for: SARS-CoV-2 antigenemia and RNAemia in association with disease severity in patients with COVID-19
Source: Sci Rep. 2024 Jun 28;14:14926. doi: 10.1038/s41598-024-65489-0 (PMC11213952; doi:10.1038/s41598-024-65489-0)
Supplement: Supplementary file 1 — Supplementary Figure 1. [file 41598_2024_65489_MOESM1_ESM.pptx]

## Slide 1
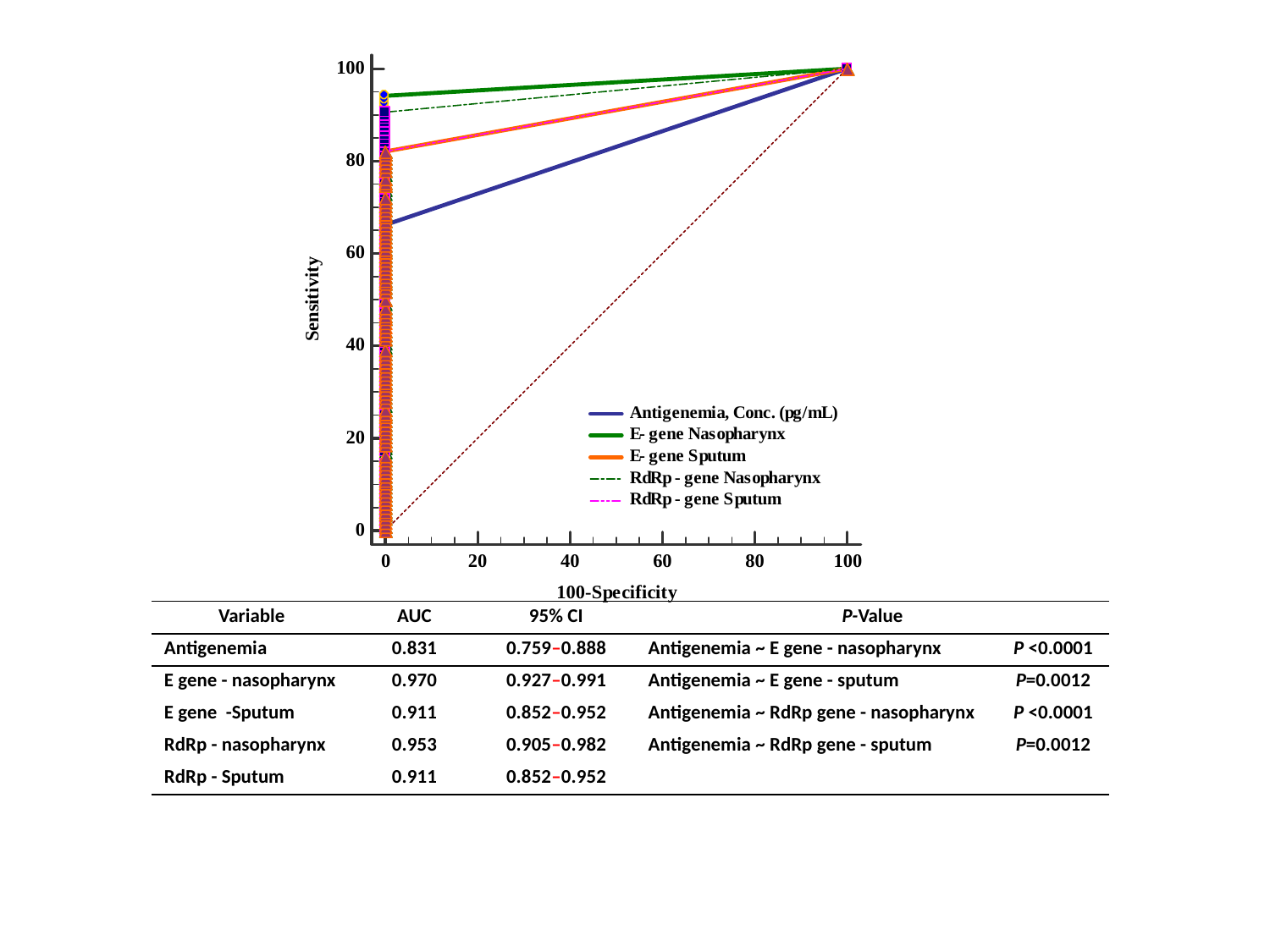

| Variable | AUC | 95% CI | P-Value | |
| --- | --- | --- | --- | --- |
| Antigenemia | 0.831 | 0.759–0.888 | Antigenemia ~ E gene - nasopharynx | P <0.0001 |
| E gene - nasopharynx | 0.970 | 0.927–0.991 | Antigenemia ~ E gene - sputum | P=0.0012 |
| E gene -Sputum | 0.911 | 0.852–0.952 | Antigenemia ~ RdRp gene - nasopharynx | P <0.0001 |
| RdRp - nasopharynx | 0.953 | 0.905–0.982 | Antigenemia ~ RdRp gene - sputum | P=0.0012 |
| RdRp - Sputum | 0.911 | 0.852–0.952 | | |
